# The light at the other end of the tunnel: heeding two decades of regeneration research to reclaim natural tooth preservation

**DOI:** 10.3389/fbioe.2026.1810083

**Published:** 2026-04-22

**Authors:** Samer H. Zaky, Rania El Backly

**Affiliations:** 1 Department of Oral and Craniofacial Sciences, School of Dental Medicine, University of Pittsburgh, Pittsburgh, PA, United States; 2 Center for Craniofacial Regeneration, School of Dental Medicine, University of Pittsburgh, Pittsburgh, PA, United States; 3 Endodontics, Conservative Dentistry Department, Faculty of Dentistry, Alexandria University, Alexandria, Egypt; 4 Tissue Engineering Laboratories, Faculty of Dentistry, Alexandria University, Alexandria, Egypt

**Keywords:** caries prevention, dental caries, dental technology, regenerative dentistry, tissue engineering, tooth preservation

## Abstract

Despite advancements in biomimetic and regenerative dentistry, contemporary prosthetic restorations, though clinically successful, remain fundamentally imperfect replications of the natural tooth’s inherent biological perfection. Crucially, while these advancements remain vital, the dental discipline has increasingly shifted from preventive focus to a reparative focus. Given that dental decay–the primary concern in a dental practice–is a highly predictable and preventable condition and that tooth loss is a largely avoidable outcome, we propose that the future of the discipline lies in transitioning focus from substitution of lost or damaged tooth parts to the preservation of what is hitherto biologically irreplaceable. Reviewing current dental practice and research, this perspective article challenges the dental community to maximize tooth longevity by acknowledging that in dentistry–more than any other medical discipline–prevention remains the cure. Our challenge invokes the ‘paradox of knowledge'–where expanded understanding unveils deeper unknown–to bring to the fore that the more we uncover the intricacies of the dental bioarchitecture, the clearer it becomes that preservation of the invaluable and irreproducible natural tooth structure, rather than its repair, is the achievable path forward. This realization warrants a paradigm “U-turn” towards the light at the starting end of the reparative tunnel. We contend that a successful shift from tooth replacement or regeneration to tooth preservation is twofold: it requires a mindful appraisal of contemporary scientific knowledge concerning the tooth mechano-biology as an unparalleled organ in the body, as well as a fundamental reexamination of the values of a profession existing to alleviate people’s suffering.

## 1 Introduction

The word *patient* stems from the Latin *patiens*, denoting suffering. From this very etymology, medicine was born as a discipline centered on alleviating human pain. Sharing the same humanitarian mission, dentistry evolved from a basic medico-mechanical craft of extraction into a professionalized branch dedicated to restoring the intricate form and function of the tooth (Guerini, 2025; Suddick and Harris, 1990). Today, as the profession continues to evolve, modern prosthetics remain imperfect replications of the natural tooth’s biological perfection. This shortfall has shifted scientific focus toward tooth regeneration, aiming to harness the body’s natural cellular renewal and differentiation to achieve true biological restoration (Adell et al., 2025).

## 2 Tooth engineering: conception and complexity

More than 2 decades ago, the advent of Tissue Engineering heralded tooth regeneration as a goal seemingly within reach (Zaky and Cancedda, 2009). Today, with the advancement of regenerative research and the unprecedented granularity of the information rendered accessible by today’s technology, namely, single-cell sequencing (Pagella et al., 2021) and spatial transcriptomics (Zaky et al., 2026) among others, tooth regeneration remains all the more distant (Ikeda et al., 2009; Bartolome-Lechuga et al., 2025). This paradox–where expanding knowledge reveals deeper unknowns–reflects the reality that the more we learn about the tooth the more we get the sense of how complex it is.

The complexity of a tooth lies in the harmonious dance between form and function. The developmental interfacing of six biologically and mechanically distinct tissues–enamel, dentin, pulp, cementum, periodontal ligament and bone–crafts an organ uniquely and remarkably designed to endure more than 600 chewing cycles each meal lifelong (Farooq and Sazonov, 2016). In a process of ultimate cellular arrangement, the finely tuned epithelial-mesenchymal interplay orchestrates the cells forming the various tooth tissues to multiply, specialize, and precisely coordinate the type, spatial placement, temporal nucleation, and synchronous growth of inorganic crystalline phases in the organic matrix (Scheffel, 2025). For the structural masterpiece, the uniquely composed interlocking interfaces between the tooth’s hard and soft tissues define its complexity and undergird its resistance to *bona fide* regeneration.

Since the inception of the regenerative medicine, the regeneration of teeth has been approached along two parallel paths: one seeking to restore the tooth individual tissues, the other aiming to regenerate the whole tooth organ. Inevitably, each path is marked by its own hurdles.

### 2.1 Regenerating tooth’s individual tissues

#### 2.1.1 Dentin and pulp regeneration

Structurally like bone and marrow, dentin and dental pulp form an integrated tissue-organ system. Dentin consists of mineralized collagen traversed by micron-scale tubules extending from the pulp to the dentin-enamel junction, creating a resilient, force-absorbing matrix (Kishen and Vedantam, 2007). Regenerative strategies rely on biomimetic mineralization that emulates natural protein-guided crystal nucleation and growth. While such approaches can reconstitute mineralized collagen resembling dentin, recreating its tubular architecture and complete functional properties remains a significant regenerative challenge (Dong et al., 2024).

Pulp regeneration seeks to restore vascularized, innervated tissue to fill debrided or root-canal–treated spaces biomimetically. Strategies include transplantation of dental pulp stem cells, which can generate pulp-like tissue (Shi et al., 2025; Astudillo-Ortiz et al., 2023) and even support sensory nerve regeneration (Nakashima and Tanaka, 2024). Alternatively, homing-based approaches recruit endogenous periapical stem cells into the pulp space to drive *in situ* regeneration (He et al., 2017; Alqahtani et al., 2018). It is imperative to underline that a tissue regenerated (or rather repaired) yet still lacking complete vascularization, innervation, immune cell populations and odontoblast-tubule interface, is aptly referred to, by consensus, as “pulp-like.”

#### 2.1.2 Periodontium regeneration

The periodontal ligament (PDL) anchors teeth to alveolar bone and serves as a reservoir of mesenchymal stem cells capable of regenerating the PDL, cementum, and bone (Chamila Prageeth Pandula et al., 2014). Regenerative strategies include stem cell transplantation (Vaquette et al., 2019), scaffold-guided tissue engineering (Virvescu et al., 2025), and emerging 3D bioprinting to recapitulate ligament architecture (Yao et al., 2022). Persistent challenges involve sustaining cell viability in complex constructs, integrating vascular and neural networks, and designing biomaterials that harmonize with healing while balancing anti-inflammatory and osteoconductive functions (Wang et al., 2024). Cementum regeneration, as part of PDL engineering, focuses on inducing neo-cementum formation on root dentin, with preclinical models demonstrating promising results (Zhao et al., 2004).

#### 2.1.3 Enamel regeneration

As the body’s hardest tissue, enamel remains the hardest to regenerate (Baranova et al., 2020). Current strategies use biomimetic and molecular engineering to mimic natural biomineralization by regulating calcium–phosphate deposition (Dong et al., 2024). Autologous biomolecules and amelogenin-derived peptides can induce enamel-like crystal growth yet engineered layers lack native mechanical strength (Moradian-Oldak, 2012; Sedek and Holiel, 2025; Li et al., 2024). Although generating ameloblast-like cells from stem cells remains a key frontier (Zhu et al., 2025), enamel regeneration is still largely experimental (Alnasser et al., 2024; Sui et al., 2025).

The true challenge in regenerating a tooth’s individual tissue lies not in the mere synthesis of the tissue *per se*, but in the reconstruction of the intricate interfaces that unify the tissues into an organ (Zaky and Cancedda, 2009; Angelopoulos, 2026). Natural development is a simultaneous, integrated process resembling a bottom-up 3D printing where layered connections are embedded incrementally rather than joined as afterthought components. Consequently, the complex biological boundaries of the tooth–including the dentino-enamel junction, Sharpey’s fibers anchoring into cementum and bone, and the odontoblastic processes infiltrating dentin–remain an unmet front in successful regeneration.

### 2.2 Tooth organ-level bioengineering

The ultimate ambition of dental regenerative medicine is not merely to repair a fragment or fill a void, but to recreate an entire living tooth’s exquisite architecture and intricate function. Recent advances have brought this vision closer to reality, revealing that stem cells can self-organize and recapitulate nature’s original blueprint.

Whole-tooth regeneration begins with the reawakening of its developmental couplet: the epithelium and the mesenchyme. The duet is the foundation for tooth regeneration strategies regardless of our success elucidating their reciprocal signaling that embryonically orchestrates tooth formation (Thesleff, 2018; Yelick and Sharpe, 2019).

Focusing on the epithelium, recent work in murine ectopic models has identified Pitx2 as a pivotal transcriptional marker of dental epithelial identity (Hu et al., 2024). Both primary and induced Pitx2^+^ cells differentiated into ameloblasts when co-cultured with dental mesenchyme and, even without exogenous growth factors, generated functional tooth structures. Pitx2 thus emerges as a signature gene and a molecular guide for directing embryonic stem cells toward a dental epithelial lineage.

In a separate model, tooth-bud regeneration in rabbits produced structures resembling a post-Bell-stage tooth germ, demonstrating promising morphological and biological features that are still preliminary for a fully developed post-Bell-stage (Sadrabad et al., 2024). Similarly, researchers have successfully achieved orthotopic regeneration and development of whole teeth in pigs by allogeneic reassociation and transplantation of tooth germ cells. For suppression of immune rejection, bone marrow mesenchymal stem cells were injected systemically in addition to local aspirin administration. While still in its infancy, this approach constitutes a notable advancement towards possible clinical realization of whole tooth engineering in large mammals (Wu et al., 2019).

Deciphering the epithelial-mesenchymal crosstalk orchestrating crown and root morphogenesis remains a fundamental barrier. While molecular signaling determines tooth identity, even overriding anatomical position (Ikeda et al., 2009), the specific transcriptomic codes–whether spatial, temporal, or age-dependent–that distinguish intricate dental features remain elusive. Defining these complex molecular signatures continues to be the ultimate frontier of whole-tooth regeneration.

On the root level, pulp and periodontal stem cells have shown the remarkable capacity to self-assemble into root-like organoids, mirroring the organization of natural root tissues and demonstrating the inherent biological intelligence embedded within postnatal human dental stem cells (Calabrese et al., 2024). These findings indicate that postnatal dental stem cells retain tooth-forming potential, capable of partial self-organization under the right conditions.

It is intriguing to acknowledge that the position and morphology of each tooth, much like the architecture of a cathedral (Hilloowala and Kanth, 2007), serve as essential pillars in craniofacial biomechanics (Benazzi et al., 2016). A striking example is found in the human upper molars, whose buccal and palatal roots distribute occlusal forces respectively between the body of the maxilla vertically and its palatine process horizontally (Hilloowala and Kanth, 2007). Yet, it remains provocative that both crown and root architecture–whether a single tapering root or a multirooted complex–are predetermined *in utero*, long before the tooth experiences any occlusal load. This highlights that genetic and epigenetic signaling cues, rather than mechanical stress, govern root number and configuration. Dissecting these cues that likely emerge from the same epithelial-mesenchymal dialogue shaping the crown may be the key to regenerating a root that looks natural and anchors naturally.

Today, Kyoto University launched a clinical trial targeting USAG-1 to treat congenital tooth agenesis (anodontia) which marks a milestone in regenerative dentistry (Takahashi et al., 2024). Mice anti-USAG-1 neutralizing antibodies were found to rescue developmentally arrested tooth germs programmed to certain tooth types (Pagella et al., 2021). Building on this discovery, USAG-1 inhibitors and antibodies have restored tooth formation in preclinical models of hypodontia and oligodontia (Takahashi et al., 2024). This progress highlights a promising new class of molecular therapeutics, though its transition from preclinical models to clinical reality requires further validation and it may be limited to early developmental stages or specific cases of congenital agenesis rather than adult tooth regeneration (Ravi et al., 2023).

## 3 The tooth: an organ apart

Given the aforementioned challenges that continue to detain the realization of tooth regeneration, we feel compelled to draw the attention of the dental community to two fundamental aspects that underscore the uniqueness of teeth among the other organs of the body. The first concerns their longevity, and the second, the predictability of their pathology.

### 3.1 From womb to tomb: the tooth’s biological finality

Regeneration is an intrinsic property of healthy tissues, driven by cycles of cell division, differentiation, and death. Its pace varies with tissue type, vascularity, and cellular composition (Adell et al., 2025).

Owing to its structural complexity, the tooth is not a single regenerative unit; its six biologically and mechanically diverse tissues display distinct capacities for renewal. In the root, the periodontal ligament and alveolar bone are highly cellular and vascularized, enabling active remodeling. Cementum, by contrast, is sparsely cellular and poorly vascularized, exhibiting limited renewal, though repair may arise from progenitor cells in the adjacent ligament (Nanci, 2012).

At the tooth’s *core*, or heart, the dental pulp contains diverse cell populations with different regenerative roles. Perivascular stem/progenitor populations are central drivers of pulp regenerative capacity by self-renewal (Dissanayaka et al., 2025) while abundant pulp fibroblasts support immune defense and neurovascular maintenance (Alvarez-Vasquez and Castaneda-Alvarado, 2022). In contrast, odontoblasts lining the dentin-pulp interface persist for the life of a healthy tooth (Couve et al., 2013). The “amazing odontoblasts” (Couve et al., 2013) show notable resistance to apoptosis and limited regenerative capacity that, though mechanistically unclear, are paralleled only by central neurons (Santos et al., 2025). Although odontoblasts at the pulp–dentin interface can generate reparative dentin in response to mild injury, the bulk dentin beneath the enamel that is lost to caries or fracture does not regenerate and remains permanently depleted.

Enamel is even more limited. In stark contrast to rodents, once human enamel is complete, its architect ameloblasts are lost, leaving enamel incapable of self-repair after decay or mechanical damage (Warshawsky et al., 1981). Notably, the enduring strength of the stiff enamel is function of its underlying resilient dentin that, by its turn, is function of tubular architecture, elastic modulus gradients, and fluid dynamics within the tubulo-canalicular system out of a moisturizing vital pulp (Kishen and Vedantam, 2007). At its core, the tooth’s remarkable resilience arises from, not despite, its soft heart.

In essence, within the human body, enamel and dentin are irreplaceable; once damaged, they are lost forever. This biological finality defines the tooth’s singularity: its mineralized tissues are inherently designed to endure a lifetime, from womb to tomb.

Despite this intrinsic endurance, the current clinical reliance on “medico-mechanical” interventions fails to honor such biological merit. Addressing the reality that, in today’s societies, old age has become notorious for the use of dentures, Simon and Giannobile boldly explicate “*Today, edentulism is no longer a consequence of age but is one of structural injustice”* (Simon and Giannobile, 2021). Affirmatively, the fact that the highest accolade a dentist bestows upon a tooth replacement is that it “looks natural” serves as the profession’s implicit recognition that there is no higher justice a dentist can offer than keeping a healthy natural tooth untouched; and that there is no healthier smile the profession can advertise for its service than the one coaxed by the tooth’s irreproducible natural tissues.

### 3.2 Tooth decay is predictable and preventable

The evolution of dental practice, under various professional titles over the centuries, has been closely and inevitably intertwined with the history of tooth decay (Guerini, 2025; Suddick and Harris, 1990). Ironically, tooth decay, the most prevalent health condition across countries of all income levels (WHO. Global oral health status report, 2022), is both predictable and preventable. Unlike acute medical conditions that may present unpredictably, dental caries develops through well-characterized biological processes and identifiable risk factors. Although it remains a multifactorial and probabilistic disease rather than a strictly predictable one, its progression can often be anticipated and interrupted (Heng, 2016).

**FIGURE 1:**
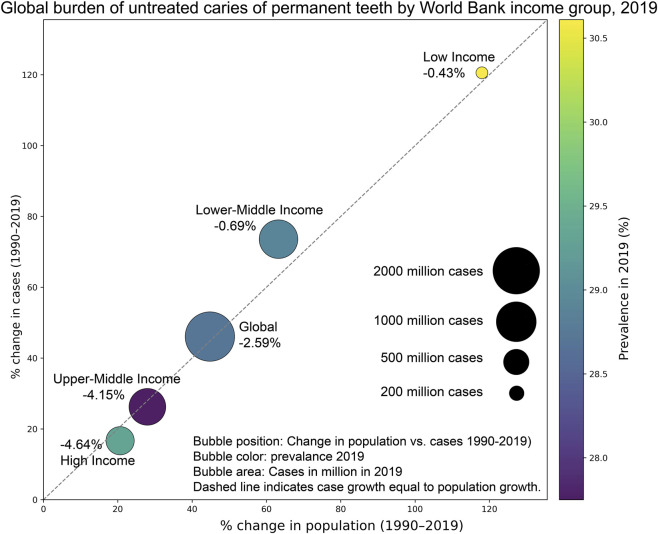
Multi-encoding bubble chart illustrating the global burden of untreated dental caries in permanent teeth across World Bank income groups (Low, Lower-middle, Upper-middle, High) and globally (Global). Values annotated above each bubble represent the percentage change in prevalence over the same period (1990–2019). The figure shows that absolute case numbers are highest in lower- and upper-middle-income countries, where population growth has been substantial. While higher-income countries show slower case growth relative to demographic change, low- and lower-middle-income settings, increases in caries cases closely track or exceed population growth, indicating limited progress in prevention. Despite these differences, prevalence remains similarly high across all income groups, underscoring the persistent and widespread global burden of dental decay. Figure created by Microsoft Copilot and refined by authors as summary of Table 4 in the WHO Global Oral Health Status Report, 2022, p. 34 (Heng, 2016).

According to a 2025 report on the global burden of oral conditions from 1990 to 2021, combined global age-standardized prevalence of oral conditions such as untreated caries, severe periodontitis, edentulism, and other oral disorders was 45,900 per 100,000 population in 2021, affecting 3.69 billion people globally (GBDOD, 2025). Unfortunately, relatively little changes in prevalence and burden of these conditions have occurred over these 3 decades (Figure 1). Although the global oral health burden is influenced by many factors such as socioeconomic disparities, demographics, and population aging, emphasis on the crucial importance of prevention must be made as we aim to change these trends in the future.

As science strives to recreate what dental materials cannot yet replicate and what current research has yet to authentically regenerate (Baranova et al., 2020), the message remains clear and humble: until such dream is realized, our foremost duty remains to preserve the tooth’s natural structures. Departing from the usual emphasis on novelty, which in tooth regeneration falls short, this writing rather megaphonically recalls Markley’s old but timeless outcry from JADA 1951, *“The loss of even part of a human tooth should be considered a serious injury and that dentistry’s goal should be to preserve healthy, natural tooth structure.”* (Markley, 1951).

## 4 Reclaiming prevention as the light at the other end of the reparative tunnel

“Tunneling” refers to the promise of progress manifested by the optimistic rush toward an alluring goal (Heath, 2020). In dentistry, few things capture that spirit more than today’s explosion of technological innovation. CAD-CAM, 3D printing, and AI-driven digital design herald unprecedented diagnostic and manufacturing speed and accuracy; intraoral scanners–cherished by dentists and patients alike–render traditional impressions history; chairside crowns and dentures are crafted with biomimetic artistry; CBCT reveals root fractures and accessory canals with unmatched clarity; dental implants aided by robotics are now placed with precise predictability; and multi-appointment procedures are now executed in a single visit with all-time economic efficiency.

Excitingly, regenerative therapies, largely based on cell-homing strategies, are bringing dentistry closer to restoring lost natural tissues. For instance, regenerative endodontics targeting revitalization is revolutionizing endodontic practice. The changing paradigm of partial or full pulpotomies (Vital Pulp Therapy) can now preserve teeth that would otherwise be devitalized and at increased risk for future loss. It is interesting that while the prospect of regenerating new dentin-pulp tissues is exciting, studies are now focusing on whether these regenerative strategies can indeed improve the structural integrity of necrotic teeth and whether these teeth can function similar to their natural counterparts (Nosr et al., 2025; Zhou et al., 2017). While these regenerative approaches have been shown to provide high healing rates, functional tooth retention, increased root volumes and to significantly influence root development, it remains to be seen if these outcomes are sustainable with longer term follow-up. Given the primarily mechanical function of teeth, durable outcomes depend on both biological repair and structural integrity–considerations that are strongly supported, upstream, through prevention.

If a dental clinic treating preventable decay is paralleled to an endocrinology unit managing diabetes, then contemporary restorative, prosthetic, and regenerative interventions may be viewed as analogous to the sophisticated rehabilitative measures required after a gangrenous amputation–clinically essential, technologically impressive, and often transformative, yet ultimately addressing the consequences of a condition that was, in principle, preventable. The celebration of an advanced “biomimetic” replacement should not divert the professed focus from preventing the amputation in the first place–or, in dentistry, “the loss of even part of a human tooth” (Markley, 1951).

It’s no surprise that much of the excitement in clinical dentistry concentrates in the restorative and prosthetic realm, where rapid technological progress can be readily translated into tangible outcomes for patients. At the same time, the economic structure of care may sometimes reward procedures more than prevention, risking an unintended imbalance in priorities. The allure within the tunnel of high-tech solutions and financial return can blind the attention to the very path being pursued or, more importantly, distract from questioning whether the travel is in the right direction.

**FIGURE 2:**
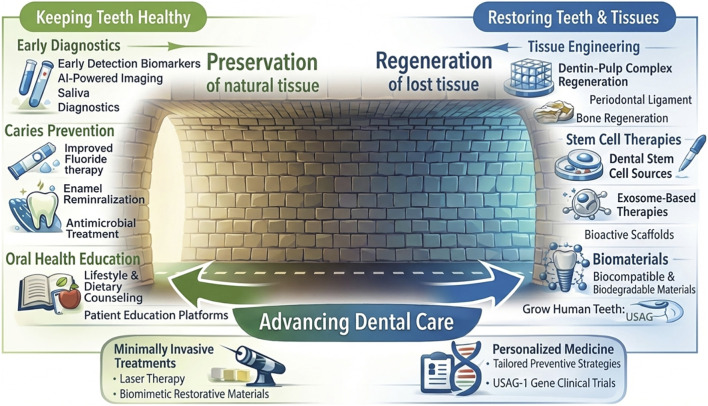
Conceptual diagram illustrating the dual paths toward advancing dental care. One end of the tunnel highlights how science is moving closer to regenerating and restoring lost tooth tissues through stem cell and tissue engineering therapies, bioactive and biomimetic scaffolds, and ultimately the emerging potential of gene therapy to induce the growth of an entirely new set of teeth. In contrast, the light behind us at the other–or starting - end of the tunnel emphasizes that maintaining oral health remains the only true way to preserve natural tooth structure. This is achieved through improved early caries diagnostics, including AI-powered tools and salivary biomarkers; enhanced caries-prevention strategies that promote enamel remineralization and antimicrobial control; and the development of advanced, interactive patient-education platforms to improve oral health awareness and prevention. Figure generated with several AI platforms and refined by authors.

This discussion is by no means a dismissal of technology. Technological advances have undeniably improved patients’ care through enhanced clinical precision and diagnostic predictability, including the early detection of caries and the interpretation of subtle radiographic changes (Rudol et al., 2025). However, unlike medical specialties that treat disease after it strikes (Suddick and Harris, 1990), dentistry primarily manages a condition where prevention remains the true cure. Since current regenerative therapies yield imperfect replicas of a natural tooth, the dental profession must invest in prevention with the same, if not more, vigor it devotes to innovation; ensuring that the salient advancement of technology does not distract from the silent success of a disease that never happened–the brighter light at the other end of the tunnel (Figure 2).

This call is particularly urgent for the burgeoning new dentists out of dental schools. Instead of technology tunneling, dental education must strike a mindful balance between teaching dental techniques in parallel to the irreproducibility of natural teeth, with an unwavering emphasis on inculcating prevention. Ruby et al. point out while wondering whether G.V. Black–who once championed the ideas of “extension for prevention” and “extension for convenience” in the 1970s–would be surprised to see that, despite decades of technological advancements such as caries detectors, acid etchants, glass ionomers, and composites that support minimally invasive care, most dental schools still prioritize a restoration-centered curriculum over a preventive education (Ruby et al., 2010).

Truth be told, this challenge is just as real for dental students as it is for practicing dentists. Students enter the dental school to gain hands-on experience by performing procedures, not by avoiding them. In the United States, these students can graduate with encumbering debt and must navigate a system where insurance companies often dictate treatment decisions based on coverage rather than need (Gaffney et al., 2025). In this context, it becomes a steep uphill battle to instill the mindset that doing less is better for the patient; that the silence of healthy teeth, with no need for intervention, is in fact the sound of their own success.

### 4.1 Advancing dentistry

If dentistry is an art, it is because it strives to replicate beauty–a beauty it tends to, ironically, overlook in its natural form. In a world where cosmetic enhancement and technological efficiency reign supreme, we often miss the wonders and beauty of what we already possess: our priceless healthy natural teeth. This is not just a technical issue, but a cultural one. Dental education, insurance incentives, and reparative technology tunneling have collectively diverted the dental practice from its conservative roots and decoyed the profession from the preventive service it avowedly exists for (Simon and Giannobile, 2021; Niederman et al., 2017; Vujicic, 2018). Hippocrates best distills the essence of this challenge in his 400 BC timeless wit “*The best medicine of all is to teach people how not to need it*”. Together, can we advance dentistry back to 400 BC?

## Data Availability

Publicly available data were analyzed in this study. This data can be found here: https://www.who.int/publications/i/item/9789240061484.
